# O Valor Preditivo do Escore CHA2DS2-VASc no Escore Syntax Residual em Pacientes com Infarto do Miocárdio com Supradesnivelamento do Segmento ST

**DOI:** 10.36660/abc.20210670

**Published:** 2022-07-07

**Authors:** Ali Kemal Kalkan, Serkan Kahraman, Yalcin Avci, Umit Bulut, Recep Gulmez, Ayse Beril Turkyilmaz, Mehmet Erturk

**Affiliations:** 1 University of Health Sciences Mehmet Akif Ersoy Thoracic and Cardiovascular Surgery Center Training and Research Hospital Istanbul Turquia University of Health Sciences, Mehmet Akif Ersoy Thoracic and Cardiovascular Surgery Center, Training and Research Hospital, Istanbul – Turquia

**Keywords:** Infarto do Miocárdio com Supradesnível do Segmento ST, Intervenção Coronária Percutânea, Fibrilação Atrial

## Abstract

**Fundamento:**

O escore CHA2DS2-VASc está associado a desfechos clínicos adversos em pacientes com doença cardiovascular. O escore Syntax residual (residual Syntax score — rSS) é uma ferramenta de pontuação que tem valor prognóstico em pacientes com infarto agudo do miocárdio com supradesnivelamento do segmento ST (IAMCSST).

**Objetivos:**

Este estudo objetivou investigar o valor preditivo do escore CHA2DS2-VASc para o rSS em pacientes com IAMCSST.

**Métodos:**

Foram avaliados 688 pacientes consecutivos com IAMCSST submetidos à intervenção coronária percutânea. Além do escore CHA2DS2-VASc, variáveis demográficas e clínicas de referência foram analisadas. Os pacientes foram divididos em dois grupos: grupo 1 – indivíduos com rSS até 8 (509 pacientes); grupo 2 – aqueles com rSS acima de 8 (179 pacientes). Valores p<0,05 foram considerados estatisticamente significativos.

**Resultados:**

O escore CHA2DS2-VASc foi maior no grupo 2 [1 (0–2); 1 (1–3), p<0,001] comparado ao grupo 1. A incidência de hipertensão [151 (29,7%); 73 (40,8%), p=0,006], idade ≥75 anos [18 (3,5%); 21 (11,7%), p<0,001], diabetes mellitus [85 (16,7%); 50 (27,9%), p=0,001] e doença vascular [12 (2,4%); 11 (6,1%), p=0,029] foi maior no grupo 2. Na análise de regressão logística multivariada, o escore CHA2DS2-VASc (odds ratio — OR=1,355; intervalo de confiança de 95% — IC95%=1,171–1,568; p<0,001), idade ≥75 anos [OR=3,218; IC95%=1,645–6,295; p=0,001] e diabetes mellitus [OR=1,670; IC95%=1,091–2,557; p=0,018] foram preditores independentes de rSS elevado. A análise da curva receiver-operating characteristic demonstrou o bom valor preditivo do escore CHA2DS2-VASc para rSS elevado com valor de corte de 1,5 (área sob a curva/area under the curve — AUC= 0,611, IC95%=0,562–0,659, p<0,001).

**Conclusões:**

O escore CHA2DS2-VASc tem valor preditivo para rSS em pacientes com IAMCSST. Além disso, o escore CHA2DS2-VASc foi um preditor independente de rSS mais alto.

## Introdução

O infarto agudo do miocárdio com supradesnivelamento do segmento ST (IAMCSST) ainda é a principal causa de aumento das taxas de morbidade e mortalidade em doenças cardiovasculares.^1^ Por esse motivo, os determinantes prognósticos de eventos cardiovasculares adversos nessa população são estudados em diversos ensaios randomizados e registros clínicos. A gravidade da doença arterial coronariana está relacionada com a maior carga aterosclerótica coronariana, resultando em um pior prognóstico, especialmente em pacientes com IAMCSST.^2^

O escore Syntax residual ( *residual Syntax score* — rSS) é um sistema de pontuação que reflete a aterosclerose coronariana obstrutiva após a realização de intervenção coronária percutânea (ICP) da lesão culpada. Demonstrou-se que o aumento do rSS (>8) teve valor prognóstico para o infarto do miocárdio (IM) e para mortalidade em 1 ano em pacientes com síndrome coronariana aguda de alto risco.^2^

A doença arterial coronariana pode surgir acompanhada de várias comorbidades. Idade, gênero, hipertensão e diabetes mellitus são alguns dos fatores de risco relacionados à evolução da aterosclerose coronariana.^3^ A maioria dos pacientes com doença arterial coronariana apresenta pelo menos um de seus fatores de risco, ou ainda uma combinação desses fatores, resultando em aumento da carga aterosclerótica coronariana.^4 , 5^

O escore CHA_2_DS_2_-VASc é descrito primeiramente para determinar a atividade aterotrombótica na fibrilação atrial.^6^ Estudos anteriores revelaram que o escore CHA_2_DS_2_-VASc estava associado a desfechos clínicos adversos em pacientes com doença cardiovascular. O escore CHA_2_DS_2_-VASc foi associado à gravidade da doença arterial coronariana^7^ e à mortalidade por todas as causas em pacientes com IAMCSST.^8^ Contudo, até onde sabemos, a relação entre o escore CHA_2_DS_2_-VASc e o rSS ainda não foi estudada. Este estudo objetivou investigar o valor preditivo do escore CHA_2_DS_2_-VASc para o rSS em pacientes com IAMCSST.

## Métodos

Seiscentos e oitenta e oito (688) pacientes consecutivos com IAMCSST submetidos à ICP entre 2017 e 2020 foram incluídos neste estudo observacional retrospectivo.

Os critérios de inclusão foram: (A) dor torácica típica por mais de 20 minutos, (b) supradesnivelamento do segmento ST em pelo menos duas derivações contíguas e (c) tratamento com ICP primária. Foram excluídos do estudo pacientes tratados apenas com terapia médica ou submetidos à cirurgia de revascularização do miocárdio. Além disso, pacientes com histórico de revascularização coronariana com intervenção percutânea ou cirúrgica também foram excluídos da pesquisa.

O estudo foi aprovado pelo comitê de ética local do Istanbul Mehmet Akif Ersoy Thoracic and Cardiovascular Surgery Training and Research Hospital em maio de 2020 (nº 2020/28).

Parâmetros demográficos e clínicos foram coletados do banco de dados do hospital. Foram realizadas análises bioquímicas, incluindo hemograma completo, creatinina sérica, glicose, colesterol e eletrólitos. Parâmetros clínicos do escore CHA_2_DS_2_-VASc foram avaliados.

Insuficiência cardíaca congestiva foi definida como sinal ou sintoma de insuficiência cardíaca ou evidência objetiva de fração de ejeção reduzida (<%40). A hipertensão foi estabelecida como pressão arterial de repouso >140/90 mmHg em pelo menos duas ocasiões ou tratamento com anti-hipertensivos. A diabetes mellitus foi caracterizada como glicemia de jejum de pelo menos 8 horas >125 mg/dL ou uso prévio de antidiabético oral e/ou insulinoterapia. A doença vascular foi definida como histórico prévio de IM, doença arterial periférica ou placa aórtica. Além disso, o índice de IAMCSST não foi incluído neste sistema de pontuação.

Angiografia coronariana e ICP foram realizadas imediatamente por meio de acesso femoral ou radial em cada paciente. Dois cardiologistas independentes e experientes avaliaram as imagens angiográficas coronarianas individualmente para calcular a gravidade da doença arterial coronariana.

O rSS foi determinado com base na obstrução residual da artéria coronária após a realização da ICP da lesão culpada. Primeiro, as artérias coronárias foram definidas como 16 segmentos separados. Cada segmento foi avaliado e segmentos com pelo menos 50% de estenose luminal e 1,5 mm de diâmetro foram analisados. Alguns aspectos determinantes também foram examinados, como um fator de ponderação correspondente pré-especificado para cada segmento, calcificação e comprimento da lesão.

A calculadora do escore Syntax (www.syntaxscore.com) foi usada para obter o rSS de cada paciente. Em seguida, os pacientes foram divididos em dois grupos de acordo com seus valores de rSS — pontuação até 8: grupo de rSS baixo (grupo 1); pontuação acima de 8: grupo de rSS elevado (grupo 2).

### Análise estatística

A análise estatística foi feita no programa Statistical Package for the Social Sciences (IBM SPSS Statistics para Windows, IBM Corp., Armonk, Nova York, EUA). Os testes qui-quadrado de Pearson, qui-quadrado com correção de continuidade e exato de Fisher foram realizados para variáveis categóricas, quando necessário. A adequação à distribuição normal foi analisada com o teste de Kolmogorov-Smirnov.

Utilizou-se “média±desvio padrão” para variáveis com distribuição normal, “mediana (percentis 25–75)” para variáveis sem distribuição normal e “n (%)” para variáveis categóricas. As análises foram realizadas com o teste *t* para amostras independentes para comparar variáveis quantitativas com distribuição normal, enquanto o teste U de Mann-Whitney foi usado para comparar as médias entre grupos sem distribuição normal.

A análise de Spearman foi utilizada para avaliar a correlação entre o escore CHA_2_DS_2_-VASc e o rSS. Foram empregadas análises de regressão logística univariada e multivariada para avaliar preditores independentes de rSS elevado.

Foi realizada uma análise da curva *receiver-operating characteristic* (ROC) para determinar o valor ideal do escore CHA_2_DS_2_-VASc para indicar rSS elevado em termos de sensibilidade e especificidade. Valores p<0,05 foram considerados estatisticamente significativos.

## Resultados

Neste estudo, foram avaliados 688 pacientes consecutivos com IAMCSST submetidos a ICP primária. Destes, 509 tinham rSS baixo (grupo 1) e 179 tinham rSS elevado (grupo 2). A Tabela 1 apresenta as variáveis demográficas e clínicas de referência de todo o grupo de estudo. Não houve diferença entre os dois grupos em termos de gênero, tabagismo, histórico de doença pulmonar obstrutiva crônica, fração de ejeção, creatinina, leucócitos, plaquetas, colesterol total, colesterol da lipoproteína de baixa densidade, colesterol da lipoproteína de alta densidade e triglicérides.

**Tabela 1:** Variáveis demográficas e clínicas de referência dos pacientes

	Grupo de rSS baixo (n=509)	Grupo de rSS elevado (n=179)	p
Idade (anos)	54±11	59±11	**<0,001**
Gênero (feminino), n (%)	88 (17,3)	40 (22,3)	0,135
Fumante, n (%)	245 (48,1)	76 (42,5)	0,190
Dislipidemia, n (%)	49 (9,6)	28 (15,6)	**0,028**
DPOC, n (%)	14 (2,8)	11 (6,1)	0,064
Fração de ejeção (%)	50 (40-55)	45 (40-55)	0,154
Creatinina (mg/dL)	0,85 (0,74-1,0)	0,85 (0,72-1,05)	0,809
Hemoglobina (g/dL)	14,8 (13,4-15,8)	14,3 (13,0-15,3)	**0,009**
Leucócitos x 103/mm^3^	11,9 (9,61-14,07)	12,3 (9,6-15,2)	0,178
Plaquetas x 103/mm^3^	261 (224-317)	264 (224-320)	0,849
Glicose (mg/dL)	132 (109-181)	155 (121-230)	**<0,001**
Colesterol total (mg/dL)	198,5±42,3	200±45,4	0,689
LDL-C (mg/dL)	120±37	122±39	0,615
HDL-C (mg/dL)	40 (33,5-46)	41 (35-48)	0,068
Triglicérides (mg/dL)	181 (118-258)	160 (111-235)	0,139
Lesão culpada, n (%)			
DAE	274 (53,8)	64 (35,8) ^*^	**<0,001**
ACX	79 (15,5)	36 (20,1)	
ACD	156 (30,6)	79 (44,1) ^†^	
Escore CHA_2_DS_2_-VASc	1 (0-2)	1 (1-3)	**<0,001**

^*^: menor que o grupo de rSS baixo, †: maior que o grupo de rSS baixo. rSS: escore Syntax residual; DPOC: doença pulmonar obstrutiva crônica; LDL-C: colesterol da lipoproteína de baixa densidade; HDL-C: colesterol da lipoproteína de alta densidade; DAE: descendente anterior esquerda; ACX: artéria circunflexa; ACD: artéria coronária direita.

A idade média do grupo 2 foi maior que a do grupo 1. A incidência de dislipidemia foi menor no grupo 1. O grupo 2 apresentou níveis menores de hemoglobina e maiores de glicose. Com relação ao vaso culpado, a incidência da artéria descendente anterior esquerda foi maior no grupo 1, enquanto a da artéria coronária direita (ACD) foi maior no grupo 2. O valor mediano do escore CHA_2_DS_2_-VASc foi mais alto nos pacientes com rSS elevado em comparação aos pacientes com rSS baixo.

A Tabela 2 traz a comparação das variáveis no sistema do escore CHA_2_DS_2_-VASc entre os grupos. Não houve diferença na incidência de insuficiência cardíaca congestiva, histórico de acidente vascular cerebral/ataque isquêmico transitório ou tromboembolismo, idade (65–74 anos) e gênero entre os grupos. A incidência de hipertensão, idade ≥75 anos, diabetes mellitus e doença vascular foi maior no grupo 2 em relação ao grupo 1. Além disso, o número de pacientes com escore CHA_2_DS_2_-VASc de 0 foi mais alto no grupo 1, enquanto o número de pacientes com escore CHA_2_DS_2_-VASc de 4 e 5 foi maior no grupo 2 ( Tabela 3 ).

**Tabela 2:** Comparação de variáveis do sistema de escore CHA2DS2-VASc em pacientes com rSS baixo e elevado

	Grupo de rSS baixo (n=509)	Grupo de rSS elevado (n=179)	p
Insuficiência cardíaca congestiva/disfunção do VE, n (%)	150 (29,5)	60 (33,5)	0,312
Hipertensão, n (%)	151 (29,7)	73 (40,8)	**0,006**
Idade ≥75 anos, n (%)	18 (3,5)	21 (11,7)	**<0,001**
Diabetes mellitus, n (%)	85 (16,7)	50 (27,9)	**0,001**
Histórico de AVC/AIT ou tromboembolismo, n (%)	1 (0,2)	0 (0)	0,740
Doença vascular, n (%)	12 (2,4)	11 (6,1)	**0,029**
65–74 anos, n (%)	76 (14,9)	33 (18,4)	0,162
Gênero (feminino), n (%)	88 (17,3)	40 (22,3)	0,135

*rSS: escore Syntax residual; VE: ventrículo esquerdo; AVC: acidente vascular cerebral; AIT: ataque isquêmico transitório.*

**Tabela 3:** Comparação dos grupos quanto ao número de pacientes para cada escore CHA2DS2-VASc

	Grupo de rSS baixo (n=509)	Grupo de rSS elevado (n=179)	p
Escore CHA_2_DS_2_-VASc: 0, n (%)	195 (38,3)	44 (24,6)	**0,001**
Escore CHA_2_DS_2_-VASc: 1, n (%)	149 (29,3)	47 (26,3)	0,442
Escore CHA_2_DS_2_-VASc: 2, n (%)	97 (19,1)	42 (23,5)	0,207
Escore CHA_2_DS_2_-VASc: 3, n (%)	43 (8,4)	20 (11,2)	0,349
Escore CHA_2_DS_2_-VASc: 4, n (%)	18 (3,5)	17 (9,5)	**0,003**
Escore CHA_2_DS_2_-VASc: 5, n (%)	6 (1,2)	7 (3,9)	**0,029**
Escore CHA_2_DS_2_-VASc: 6, n (%)	1 (0,2)	2 (1,1)	0,167

*rSS: escore Syntax residual.*

Foi realizada uma análise de regressão logística e variáveis significativas encontradas na análise univariada foram incluídas na análise de regressão logística multivariada para predizer o fator de risco independente para rSS elevado. Na análise de regressão logística multivariada, o escore CHA_2_DS_2_-VASc e a ACD como lesão culpada foram considerados preditores independentes de rSS elevado ( Tabela 4 ). Ademais, na análise de regressão logística multivariada para variáveis do escore CHA_2_DS_2_-VASc, a idade ≥75 anos e a diabetes mellitus também foram preditoras independentes de rSS elevado ( Tabela 5 ).

**Tabela 4:** Análises de regressão logística univariada e multivariada fornecendo informações sobre preditores independentes de rSS elevado

	Análise univariada			Análise multivariada		
	*Odds ratio*	IC95% (Inferior-superior)	p	*Odds ratio*	IC95% (Inferior-superior)	p
Dislipidemia	1,741	1,056-2,868	0,030	1,605	0,956-2,696	0,074
DPOC	2,315	1,031-5,198	0,042	1,522	0,637-3,638	0,344
Hemoglobina	0,892	0,815-0,977	0,014	0,977	0,883-1,081	0,658
Escore CHA_2_DS_2_-VASc	1,374	1,210-1,560	<0,001	1,355	1,171-1,568	**<0,001**
ACD como lesão culpada	1,788	1,260-2,537	0,001	1,963	1,360-2,831	**<0,001**

*rSS: escore Syntax residual; IC95%: intervalo de confiança de 95%; DPOC: doença pulmonar obstrutiva crônica; ACD: artéria coronária direita.*

**Tabela 5:** Análises de regressão logística univariada e multivariada para variáveis do escore CHA2DS2-VASc para detectar preditores independentes de rSS elevado

	Análise univariada			Análise multivariada		
	*Odds ratio*	IC95% (Inferior-superior)	p	*Odds ratio*	IC95% (Inferior-superior)	p
ICC/disfunção do VE	1,207	0,838-1,737	0,312			
Hipertensão	1,633	1,146-2,325	0,007	1,296	0,888-1,892	0,179
Idade ≥75 anos	3,626	1,884-6,977	<0,001	3,218	1,645-6,295	**0,001**
Diabetes mellitus	1,933	1,295-2,887	0,001	1,670	1,091-2,557	**0,018**
AVC/AIT	0,000	0,000	1,000			
Doença vascular	2,712	1,175-6,260	0,019	2,059	0,858-4,942	0,106
65–74 anos	1,288	0,821-2,019	0,270			
Gênero (feminino)	1,377	0,905-2,095	0,136			

*rSS: escore Syntax residual; IC95%: intervalo de confiança de 95%; ICC: insuficiência cardíaca congestiva; VE: ventrículo esquerdo; AVC: acidente vascular cerebral; AIT: ataque isquêmico transitório.*

A análise da curva ROC foi feita para determinar o valor de corte ideal do escore CHA_2_DS_2_-VASc para indicar rSS elevado. Os valores mais altos de sensibilidade e especificidade combinados cruzaram a curva em 1,5 (sensibilidade de 49,2% e especificidade de 67,6%). A área sob a curva ( *area under the curve* — AUC) foi 0,611 (intervalo de confiança de 95% — IC95% 0,562–0,659; p<0,001).

A análise da curva ROC também foi realizada em homens e mulheres, separadamente. Na população masculina, o valor de corte ideal do escore CHA_2_DS_2_-VASc foi 1,5 (sensibilidade de 36,7% e especificidade de 77,0%) com AUC de 0,592 (IC95% 0,536–0,647; p=0,001). Na população feminina, o valor de corte ideal do escore CHA_2_DS_2_-VASc foi 3,5 (sensibilidade de 47,5% e especificidade de 78,4%) com AUC de 0,653 (IC95% 0,550–0,756; p=0,006).

**Figura 1:**
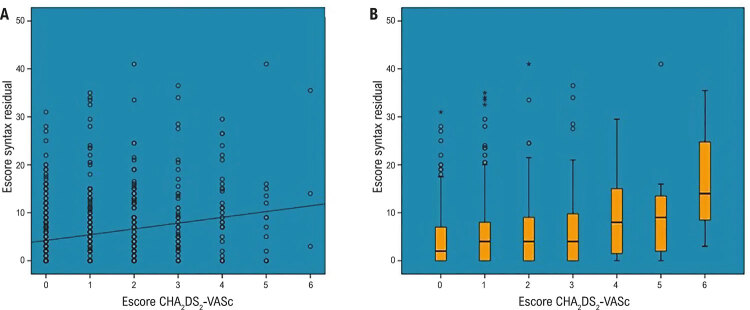
A) Correlação entre o escore CHA2DS2-VASc e o escore Syntax residual. B) Valor do escore Syntax residual para cada escore CHA2DS2-VASc.

Também ficou evidenciado que o escore CHA_2_DS_2_-VASc foi correlacionado tanto com o escore de referência quanto com o rSS. A análise de correlação de Spearman revelou uma correlação positiva entre o escore CHA_2_DS_2_-VASc e o rSS (r=0,203; p<0,001) ( Figura 1 ). Além disso, foi identificada uma correlação positiva entre o escore CHA_2_DS_2_-VASc e o rSS (r=0,234; p<0,001). Pacientes com escore Syntax de referência baixo apresentaram escore menor de CHA_2_DS_2_-VASc [1 (0–2), 1 (0–3); p<0,001] quando comparados àqueles com escore Syntax de referência intermediário ou elevado.

## Discussão

Até onde sabemos, este estudo foi o primeiro a evidenciar a associação entre o escore CHA_2_DS_2_-VASc e o rSS em pacientes com IAMCSST. Um escore CHA_2_DS_2_-VASc aumentado, idade ≥75 e diabetes mellitus foram considerados preditores independentes de rSS elevado. Adicionalmente, o escore CHA_2_DS_2_-VASc foi correlacionado com o rSS.

A doença arterial coronariana é uma condição progressiva e permanece uma causa importante para o aumento das taxas de morbidade e mortalidade em todo o mundo.^1^ Vários fatores de risco para doença arterial coronariana são bem descritos. Entre eles estão idade, diabetes mellitus, hipertensão e gênero, que demonstram a presença e extensão da aterosclerose coronariana e são aceitos como fatores de risco principais para o desenvolvimento de doenças cardiovasculares.^3^ É por esse motivo que algumas ferramentas de pontuação são descritas para determinar o risco cardiovascular e o prognóstico.

O escore CHA_2_DS_2_-VASc é um dos sistemas de pontuação mais importantes para predizer desfechos clínicos adversos em pacientes com doença cardiovascular. Foi utilizado primeiramente em pacientes com fibrilação atrial para estimar o risco de tromboembolismo.^6^ Demonstrou-se que o risco de desenvolver tromboembolismo aumenta com escores mais altos de CHA_2_DS_2_-VASc.^6^ Também foi revelado que esse escore foi um preditor útil de eventos clínicos adversos subsequentes em pacientes com síndrome coronariana aguda.^9^ O escore CHA_2_DS_2_-VASc ≥2 foi relacionado com o desfecho composto de IM, acidente vascular cerebral e morte em 3.183 pacientes com síndrome coronariana aguda.^9^ No estudo de Nof et al., cada incremento de 1-U no escore CHA_2_DS_2_-VASc foi associado a um aumento significativo de 33% no risco de mortalidade em 1.820 pacientes com insuficiência cardíaca com fração de ejeção reduzida.^10^ Além disso, o escore CHA_2_DS_2_-VASc é um preditor de mortalidade por todas as causas em pacientes com IAMCSST.^8^

À luz dos dados anteriores, o aumento da atividade trombogênica e da carga trombótica pode ser o motivo de desfechos cardiovasculares adversos em pacientes com escore alto de CHA_2_DS_2_-VASc. Esses resultados podem ser explicados por variáveis do escore CHA_2_DS_2_-VASc associadas a um processo aterotrombótico mais adiantado, como idade avançada, hipertensão, diabetes mellitus e insuficiência cardíaca.

Em um estudo de Scudiero et al., 1.729 pacientes consecutivos com síndrome coronariana aguda submetidos a intervenção percutânea foram avaliados em estudo prospetivo e o escore CHA_2_DS_2_-VASc foi relacionado à alta reatividade plaquetária.^11^ Ipek et al. também demonstraram que o escore CHA_2_DS_2_-VASc está associado a fenômenos de não reperfusão em pacientes com IAMCSST submetidos a ICP primária.^12^ Portanto, o escore CHA_2_DS_2_-VASc é uma boa ferramenta para predizer o aumento da aterotrombose.

Sabe-se que a extensão e a gravidade da doença arterial coronariana estão associadas ao estado aterotrombótico mencionado. Isso significa que uma maior atividade aterosclerótica resulta em aumento na carga aterosclerótica coronariana. Corroborando este achado, estudos anteriores revelaram a relação entre o escore CHA_2_DS_2_-VASc e a gravidade da doença arterial coronariana.

Em um estudo de Cetin et al., foram avaliados 407 pacientes consecutivos submetidos à angiografia coronariana diagnóstica e o escore CHA_2_DS_2_-VASc foi significativamente correlacionado com uma série de vasos doentes e associado à gravidade da doença arterial coronariana.^7^

Tasolar et al. analisaram 252 pacientes consecutivos com infarto agudo do miocárdio sem supradesnivelamento do segmento ST (IAMSSST) e o escore CHA_2_DS_2_-VASc foi relacionado a um escore Syntax mais elevado.^13^ Entretanto, até onde sabemos, a associação entre o escore CHA_2_DS_2_-VASc e a gravidade da doença arterial coronariana residual após a realização de ICP ainda não foi estudada.

Além de ser uma preditora de pior prognóstico, aproximadamente 40–65% dos casos de doença coronariana multiarterial são detectados em pacientes com síndrome coronariana aguda.^14 , 15^

O rSS é um sistema de classificação que determina a complexidade e a gravidade da aterosclerose coronariana após realização de ICP da lesão culpada. Foi utilizado e descrito primeiramente por meio de uma análise post-hoc do ensaio ACUITY ( *Acute Catheterization and Urgent Intervention Triage strategY* ).^2^ O rSS elevado (>8) foi um forte preditor independente de revascularização não planejada, IM, mortalidade cardíaca e em 1 ano em 2.686 pacientes com síndrome coronariana aguda de risco moderado-alto submetidos a ICP.^2^ Corroborando esse resultado, Loutfi et al. demonstraram que um rSS menor (pontuação até 8) está associado à redução em 1 ano de eventos cardíacos e cerebrovasculares adversos maiores (ECCAM), morte, IM, acidente vascular cerebral e revascularização repetida em pacientes com IAMCSST.^16^

Um resultado inesperado do grupo de subestudo do ensaio COURAGE ( *Clinical Outcomes Utilizing Revascularization and Aggressive Drug Evaluation* ) revelou que a extensão e a gravidade da obstrução anatômica das artérias coronárias tinham um valor mais preditivo para IM e morte do que o grau de isquemia.^17^ Isso reflete o valor prognóstico da carga aterosclerótica coronariana em resultados clínicos adversos. Desse modo, a importância da gravidade da doença arterial coronariana residual fica evidente.

Até onde sabemos, também foi demonstrada pela primeira vez a associação entre o escore CHA_2_DS_2_-VASc e o rSS em pacientes com IAMCSST submetidos a ICP primária. Ela pode ser a razão do aumento de desfechos cardiovasculares adversos em pacientes com IAMCSST e escore mais alto de CHA_2_DS_2_-VASc. No entanto, são necessários estudos em larga escala para investigações futuras, especialmente focados em eventos clínicos.

### Limitações do estudo

O tamanho relativamente pequeno da amostra foi a principal limitação deste estudo. A falta de dados sobre desfechos clínicos e prognóstico foi outra grande limitação. Alguns fatores de risco podem ser modificados com alterações no estilo de vida e terapia médica. Entretanto, este estudo não foi capaz de demonstrar o efeito de fatores modificadores sobre os resultados clínicos, devido ao seu delineamento retrospectivo.

## Conclusão

O escore CHA_2_DS_2_-VASc tem valor preditivo para rSS em pacientes com IAMCSST. Além disso, o escore CHA_2_DS_2_-VASc foi um preditor independente de rSS mais alto. Esse escore também foi positivamente correlacionado com a carga aterosclerótica coronariana.
