# Supplementary material for: O Valor Preditivo do Escore CHA2DS2-VASc no Escore Syntax Residual em Pacientes com Infarto do Miocárdio com Supradesnivelamento do Segmento ST
Source: Arq Bras Cardiol. 2022 Jul 7;119(3):393–9. [Article in Portuguese] doi: 10.36660/abc.20210670 (PMC9438551; doi:10.36660/abc.20210670)
Supplement: Translation

# Translation: The Predictive Value of CHA2DS2-VASc Score on Residual Syntax Score in Patients With ST Segment Elevation Myocardial Infarction

**Authors:** Ali Kemal Kalkan, Serkan Kahraman, Yalcin Avci, Umit Bulut, Recep Gulmez, Ayse Beril Turkyilmaz, Mehmet Erturk

**Affiliations:** 1 Turkey University of Health Sciences, Mehmet Akif Ersoy Thoracic and Cardiovascular Surgery Center, Training and Research Hospital, Istanbul – Turkey

**Keywords:** ST Elevation Myocardial Infarction, Percutaneous Coronary Intervention, Atrial Fibrillation

## Abstract

**Background:**

The CHA_2_DS_2_-VASc score is associated with adverse clinical outcomes in patients with cardiovascular disease. The residual Syntax score (rSS) is a scoring tool which has prognostic value in patients with ST segment elevation myocardial infarction (STEMI).

**Objectives:**

Our aim in this study is to investigate the predictive value of the CHA_2_DS_2_-VASc score on rSS in STEMI patients.

**Methods:**

A total of 688 consecutive patients with STEMI undergoing percutaneous coronary intervention were evaluated. Baseline demographic and clinical variables besides the CHA_2_DS_2_-VASc score were assessed. The patients were divided into two groups; patients with rSS of 8 or below as group 1 (509 patients) and more than 8 as group 2 (179 patients). A p-value < 0.05 was considered statistically significant.

**Results:**

The CHA_2_DS_2_-VASc score was higher in group 2 [1 (0-2); 1 (1-3), p<0.001] compared to group 1. The incidence of hypertension [151 (29.7%); 73 (40.8%), p=0.006], patients ≥75 years [18 (3.5%); 21 (11.7%), p<0.001], diabetes mellitus [85 (16.7%); 50 (27.9%), p=0.001] and vascular disease [12 (2.4%); 11 (6.1%), p=0.029] were higher in group 2. In multivariate logistic regression analysis, the CHA_2_DS_2_-VASc score (OR=1.355; 95%CI=1.171-1.568; p<0.001), age ≥75 years [OR=3.218; 95%CI=1.645-6.295; p=0.001] and diabetes mellitus [OR=1.670; 95%CI=1.091-2.557; p=0.018] were independent predictors of high rSS. The receiver-operating characteristic curve analysis demonstrated that the CHA_2_DS_2_-VASc score had good predictive value for high rSS with a cut-off value of 1.5 (area under curve (AUC): 0.611, 95% confidence interval (CI):0.562-0.659, p<0.001).

**Conclusions:**

The CHA_2_DS_2_-VASc score has a predictive value on rSS in patients with STEMI. The CHA_2_DS_2_-VASc score was also an independent predictor of higher rSS.

## Introduction

ST segment elevation myocardial infarction (STEMI) is still the leading cause of increased morbidity and mortality rates in cardiovascular diseases.^1^ Thus, prognostic determinants of adverse cardiovascular events in this population are studied in several randomized trials and clinic registries. Coronary artery disease severity is related with higher coronary atherosclerotic burden results in poorer prognosis in coronary artery disease, especially in STEMI patients.^2^

The residual Syntax score (rSS) is a scoring system which reflects obstructive coronary atherosclerosis after performing percutaneous coronary intervention (PCI) to culprit lesion. It was demonstrated that increased rSS (>8) had a prognostic value on myocardial infarction (MI) and 1- year mortality in high-risk acute coronary syndrome patients.^2^

Coronary artery disease can appear together with several comorbidities. Age, gender, hypertension, diabetes mellitus are some of these risk factors that are related with the progression of coronary atherosclerosis.^3^ Most patients with coronary artery disease had at least one risk factor of coronary artery disease and also a combination of these risk factors resulting in increased coronary atherosclerotic burden.^4 , 5^ The CHA_2_DS_2_-VASc score is firstly described to determine atherothrombotic activity in atrial fibrillation.^6^ In previous studies, it was revealed that the CHA_2_DS_2_-VASc score was associated with adverse clinical outcomes in patients with cardiovascular disease. The CHA_2_DS_2_-VASc score was found to be related with coronary artery disease severity^7^ and all-cause mortality in STEMI patients.^8^ However, to the best of our knowledge, the relationship between the CHA_2_DS_2_-VASc score and rSS has not been studied yet. Our aim in this study is to investigate the predictive value of the CHA_2_DS_2_-VASc score on rSS in STEMI patients.

## Methods

Six hundred eighty-eight (688) consecutive patients with ST segment elevation myocardial infarction (STEMI) undergoing primary percutaneous coronary intervention (PCI) from 2017 to 2020 were included in our retrospective observational study. The inclusion criteria are as follows: (a) typical chest pain for more than 20 minutes, (b) ST-segment elevation in at least two contagious leads, and (c) treatment with primary PCI. Patients who were treated with medical therapy alone or underwent coronary artery bypass grafting were excluded from the study. Additionally, patients with history of coronary revascularization with percutaneous or surgical therapy were also excluded from the study. The study was approved by the local ethics committee at Istanbul Mehmet Akif Ersoy Thoracic and Cardiovascular Surgery Training and Research Hospital in May 2020 (no:2020/28).

The demographic and clinical parameters were recorded from the hospital database. Biochemical analyses including complete blood count, serum creatinine, glucose, cholesterol and electrolytes levels were assessed. The clinical parameters in the CHA_2_DS_2_-VASc score were evaluated. Congestive heart failure was defined as signs or symptoms of heart failure or objective evidence of reduced ejection fraction (<%40). Hypertension was defined as resting blood pressure >140/90 mmHg on at least two occasions, or treatment with antihypertensive medications. Diabetes mellitus was defined as at least 8 hours fasting plasma glucose level >125 mg/dl, or the previous use of oral anti-diabetic agent and/or insulin therapy. Vascular disease was defined as history of previous myocardial infarction (MI) or peripheral arterial disease or aortic plaque. Additionally, the STEMI index was not included in this scoring system.

Coronary angiography and PCI were performed through femoral or radial access immediately for each patient. Two independent, experienced cardiologists evaluated the coronary angiographic images individually to calculate coronary artery disease severity. The residual Syntax score (rSS) was defined based on the residual coronary artery obstruction after performing the percutaneous coronary intervention (PCI) for culprit lesion. Firstly, coronary arteries were defined as 16 separate segments. Each segment was evaluated and the segment that had at least 50% of luminal stenosis and a 1.5mm diameter was assessed. Additionally, some determinant factors were evaluated, such as a pre-specified corresponding weighing factor of each segment, calcification and lesion length. The Syntax score calculator (www.syntaxscore.com) was used to obtain rSS for each patient. Then, patients were divided into two groups according to their rSS values; the patients with a score of 8 or below as the low-rSS group (group 1) and more than 8 as the high-rSS group (group 2).

### Statistical analysis

The statistical analysis was made by using the computer software Statistical Package for Social Sciences (IBM SPSS Statistics for Windows, IBM Corp., Armonk, New York, USA). Pearson chi-square, continuity-corrected chi-square and Fisher exact tests were performed for categorical variables, where appropriate. The fitness to normal distribution was analyzed with the Kolmogorov-Simirnov test. “mean±standard deviation” was used for variables with normal distribution, “median (25^th^-75^th^percentiles)” for variables without normal distribution and “n (%)” for categorical variables.

The analyses were done with an independent sample t-test for comparing quantitative variables with normal distribution, while the Mann Whitney u test was used for comparing the means between groups without normal distribution.

The Spearman analysis was used to evaluate the correlation between the CHA_2_DS_2_-VASc score and rSS. Univariate and multivariate logistic regression analyses were used to evaluate independent predictors of high residual Syntax score (rSS).

A Receiver Operating Characteristic (ROC) curve analysis was conducted to determine the optimal CHA_2_DS_2_-VASc score value to indicate high rSS in terms of both sensitivity and specificity. A p-value < 0.05 was considered statistically significant.

## Results

A total of 688 consecutive patients with ST segment elevation myocardial infarction (STEMI) who undergone primary percutaneous coronary intervention (PCI) were evaluated in this study. Of these 688 patients, 509 patients had low rSS (group 1) and 179 had high rSS (group 2). Baseline demographic and clinical variables of the entire study group were demonstrated in table 1 . There were no differences in terms of gender, smoking status, history of chronic obstructive pulmonary disease, ejection fraction, creatinine, leukocyte, thrombocyte, total cholesterol, low density lipoprotein cholesterol, high density lipoprotein cholesterol and triglyceride levels between the two groups.

**Table 1:** Baseline demographic and clinical variables of patients

	Low rSS group (n= 509)	High rSS group (n= 179)	p
Age (years)	54±11	59±11	**<0.001**
Gender (female), n (%)	88 (17.3)	40 (22.3)	0.135
Smoking, n (%)	245 (48.1)	76 (42.5)	0.190
Hyperlipidemia, n (%)	49 (9.6)	28 (15.6)	**0.028**
COPD, n (%)	14 (2.8)	11 (6.1)	0.064
Ejection fraction (%)	50 (40-55)	45 (40-55)	0.154
Creatinine (mg/dl)	0.85 (0.74-1.0)	0.85 (0.72-1.05)	0.809
Hemoglobin (g/dl)	14.8 (13.4-15.8)	14.3 (13.0-15.3)	**0.009**
Leukocyte × 103/mm^3^	11.9 (9.61-14.07)	12.3 (9.6-15.2)	0.178
Thrombocyte × 103/mm^3^	261 (224-317)	264 (224-320)	0.849
Glucose (mg/dl)	132 (109-181)	155 (121-230)	**<0.001**
Total cholesterol (mg/dl)	198.5±42.3	200±45.4	0.689
LDL cholesterol (mg/dl)	120±37	122±39	0.615
HDL cholesterol (mg/dl)	40 (33.5-46)	41 (35-48)	0.068
Triglyceride (mg/dl)	181 (118-258)	160 (111-235)	0.139
Culprit lesion, n (%)			
LAD	274 (53.8)	64 (35.8)^*^	**<0.001**
CXA	79 (15.5)	36 (20.1)	
RCA	156 (30.6)	79 (44.1)^†^	
CHA_2_DS_2_-VASc score	1 (0-2)	1 (1-3)	**<0.001**

^*^: lower than the low rSS group,^†^: higher than the low rSS group. COPD: chronic obstructive pulmonary disease; CXA: circumflex artery; HDL: high density lipoprotein; LAD: left anterior descending; LDL: low density lipoprotein; RCA: right coronary artery; rSS: residual Syntax score.

The mean age of group 2 was higher than in group 1. The incidence of hyperlipidemia was lower in group 1. While the hemoglobin level was lower in group 2, the glucose level was higher in group 2. The incidence of the culprit vessel as left anterior descending artery was higher in group 1, while the incidence of right coronary artery as a culprit vessel was higher in group 2. The median value of the CHA_2_DS_2_-VASc score was higher in patients with high rSS compared to patients with low rSS.

The comparison of variables into the CHA_2_DS_2_-VASc scoring system between groups was demonstrated in table 2 . There were no differences in the incidence of congestive heart failure, history of stroke/transient ischemic attack or thromboembolism, age (65-74 years) and sex category between groups. The incidence of hypertension, patients ≥75 years, diabetes mellitus and vascular disease was higher in group 2 compared to group 1. Additionally, the number of patients with a CHA_2_DS_2_-VASc score of 0 was higher in group 1, while the number of patients with a CHA_2_DS_2_-VASc score of 4 and 5 was higher in group 2 ( Table 3 ).

**Table 2:** Comparison of variables into the CHA2DS2-VASc scoring system between patients with low and high rSS

	Low rSS group (n= 509)	High rSS group (n= 179)	p
Congestive heart failure/LV dysfunction, n (%)	150 (29.5)	60 (33.5)	0.312
Hypertension, n (%)	151 (29.7)	73 (40.8)	**0.006**
Age ≥75 years, n (%)	18 (3.5)	21 (11.7)	**<0.001**
Diabetes mellitus, n (%)	85 (16.7)	50 (27.9)	**0.001**
History of stroke/TIA or thromboembolism, n (%)	1 (0.2)	0 (0)	0.740
Vascular disease, n (%)	12 (2.4)	11 (6.1)	**0.029**
Age 65-74 years, n (%)	76 (14.9)	33 (18.4)	0.162
Sex category (female), n (%)	88 (17.3)	40 (22.3)	0.135

*LV: left ventricle; rSS: residual Syntax score; TIA: transient ischemic attack.*

**Table 3:** Comparison of groups in terms of the number of patients for each CHA2DS2-VASc score

	Low rSS group (n= 509)	High rSS group (n= 179)	p
CHA_2_DS_2_-VASc score: 0, n (%)	195 (38.3)	44 (24.6)	**0.001**
CHA_2_DS_2_-VASc score: 1, n (%)	149 (29.3)	47 (26.3)	0.442
CHA_2_DS_2_-VASc score: 2, n (%)	97 (19.1)	42 (23.5)	0.207
CHA_2_DS_2_-VASc score: 3, n (%)	43 (8.4)	20 (11.2)	0.349
CHA_2_DS_2_-VASc score: 4, n (%)	18 (3.5)	17 (9.5)	**0.003**
CHA_2_DS_2_-VASc score: 5, n (%)	6 (1.2)	7 (3.9)	**0.029**
CHA_2_DS_2_-VASc score: 6, n (%)	1 (0.2)	2 (1.1)	0.167

*rSS: residual Syntax score.*

The logistic regression analysis was conducted and significant variables which were found in the univariate analysis were put into the multiple logistic regression analysis to predict the independent risk factor of high residual Syntax score (rSS). In the multivariate logistic regression analysis, the CHA_2_DS_2_-VASc score and RCA as a culprit lesion were found to be independent predictors of high rSS ( Table 4 ). Additionally, in the multivariate logistic regression analysis for variables into the CHA_2_DS_2_-VASc score, advanced age ≥75 years and diabetes mellitus were also independent predictors of high rSS ( Table 5 ).

**Table 4:** Univariate and multivariate logistic regression analyses providing information about independent predictors of high rSS

	Univariate analysis			Multivariate analysis		
	Odds ratio	95% CI (Lower-Upper)	p	Odds ratio	95% CI (Lower-Upper)	p
Hyperlipidemia	1.741	1.056-2.868	0.030	1.605	0.956-2.696	0.074
COPD	2.315	1.031-5.198	0.042	1.522	0.637-3.638	0.344
Hemoglobin	0.892	0.815-0.977	0.014	0.977	0.883-1.081	0.658
CHA_2_DS_2_-VASc score	1.374	1.210-1.560	<0.001	1.355	1.171-1.568	**<0.001**
Culprit lesion RCA	1.788	1.260-2.537	0.001	1.963	1.360-2.831	**<0.001**

*COPD: chronic obstructive pulmonary disease; RCA: right coronary artery.*

**Table 5:** Univariate and multivariate logistic regression analyses for CHA2DS2-VASc score variables to detect independent predictors of high rSS

	Univariate analysis			Multivariate analysis		
	Odds ratio	95% CI (Lower-Upper)	p	Odds ratio	95% CI (Lower-Upper)	p
CHF/LV dysfunction	1.207	0.838-1.737	0.312			
Hypertension	1.633	1.146-2.325	0.007	1.296	0.888-1.892	0.179
Age ≥75 years	3.626	1.884-6.977	<0.001	3.218	1.645-6.295	**0.001**
Diabetes mellitus	1.933	1.295-2.887	0.001	1.670	1.091-2.557	**0.018**
Stroke/TIA	0.000	0.000	1.000			
Vascular disease	2.712	1.175-6.260	0.019	2.059	0.858-4.942	0.106
Age 65-74 years	1.288	0.821-2.019	0.270			
Sex (female)	1.377	0.905-2.095	0.136			

*CHF: Congestive heart failure; LV: left ventricle; TIA: transient ischemic attack.*

The Receiver Operating Characteristic (ROC) curve analysis was conducted to determine the optimal CHA_2_DS_2_-VASc score cut-off value to indicate high rSS. The highest combined sensitivity and specificity values crossed the curve at 1.5 (sensitivity 49.2% and specificity 67.6%). The area under the curve (AUC) was 0.611 (95% CI:0.562-0.659, p<0.001).

The ROC curve analysis was also conducted in male and female genders, separately. In the male population, the optimal CHA_2_DS_2_-VASc score cut-off value was 1.5 (sensitivity of 36.7% and specificity of 77.0%) with the AUC of 0.592 (95% CI:0.536-0.647, p=0.001). In the female population, the optimal CHA_2_DS_2_-VASc score cut-off value was 3.5 (sensitivity of 47.5% and specificity of 78.4%) with the AUC of 0.653 (95% CI:0.550-0.756, p=0.006).

We also demonstrated that the CHA_2_DS_2_-VASc score was correlated with both baseline and residual Syntax scores. The Spearman’s correlation analysis revealed that there was a positive correlation between the CHA_2_DS_2_-VASc score and the residual Syntax score (rSS) (r:0.203, p<0.001) ( Figure 1 ). Also, there was a positive correlation between the CHA_2_DS_2_-VASc score and the residual Syntax score (rSS) (r:0.234, p<0.001). Additionally, patients with a baseline low Syntax score had a lower CHA_2_DS_2_-VASc score [1 (0-2), 1 (0-3); p<0.001] compared to patients with a baseline intermediate or high Syntax score.

**Figure 1:** A) Correlation between CHA2DS2-VASc score and residual Syntax score. B) Residual Syntax score value for each CHA2DS2-VASc score.

## Discussion

In our study, to the best of our knowledge, the association of the CHA_2_DS_2_-VASc score and rSS was demonstrated for the first time in STEMI patients. An increased CHA_2_DS_2_-VASc score, especially advanced age ≥75 and diabetes mellitus were found to be independent predictors of high rSS. Additionally, the CHA_2_DS_2_-VASc score was correlated with rSS.

Coronary artery disease is a progressive disease and still an important reason of the increased morbidity and mortality rates in worldwide.^1^ Several risk factors of coronary artery disease are well described. Age, diabetes mellitus, hypertension and gender are some of these risk factors that demonstrate the presence and extent of coronary atherosclerosis, and they are accepted as major risk factors for the development of cardiovascular disease.^3^ That is why some scoring tolls are described to determine cardiovascular risk and prognosis.

The CHA_2_DS_2_-VASc score is one of the most important scoring systems to predict adverse clinical outcomes in patients with cardiovascular disease. It was firstly used in patients with atrial fibrillation to estimate the risk of thromboembolism.^6^ It was demonstrated that the risk of development of thromboembolism increases with a higher CHA_2_DS_2_-VASc score.^6^ It was also revealed that this score was a useful predictor of subsequent adverse clinical events in patients with acute coronary syndrome.^9^ The CHA_2_DS_2_-VASc score ≥2 was found to be related with composite endpoint of myocardial infarction, stroke and death in 3183 patients with acute coronary syndrome.^9^ In a study by Nof et al., each 1-U increment in the CHA_2_DS_2_-VASc score was associated with a significant increase of 33% in mortality risk in 1820 patients with reduced ejection fraction heart failure.^10^ Additionally, the CHA_2_DS_2_-VASc score predicts all-cause mortality in patients with ST segment elevation myocardial infarction (STEMI).^8^

In light of the foregoing data, increased thrombogenic activity and thrombotic burden may be the reason for adverse cardiovascular outcomes in patients with a high CHA_2_DS_2_-VASc score. These results can be explained by variables of the CHA_2_DS_2_-VASc score which are associated with a higher atherothrombotic process, such as advanced age, hypertension, diabetes mellitus and heart failure. In a study by Scudiero et al., 1729 consecutive patients with acute coronary syndrome undergoing percutaneous treatment were evaluated in a prospective study and the CHA_2_DS_2_-VASc score was found to be related with high platelet reactivity.^11^ Ipek et al. also showed that the CHA_2_DS_2_-VASc score is associated with no-reflow phenomena in STEMI patients who underwent primary percutaneous coronary intervention (PCI).^12^ As a result, the CHA_2_DS_2_-VASc score is a good tool to predict increased atherothrombosis.

It is well known that the extent and severity of coronary artery disease is associated with the mentioned atherothrombotic status. It means higher atherosclerotic activity results in increased coronary atherosclerotic burden. Supporting this, in previous studies, the relationship between the CHA_2_DS_2_-VASc score and coronary artery disease severity was revealed.

In a study by Cetin et al., 407 consecutive patients who underwent diagnostic coronary angiography were evaluated, and the CHA_2_DS_2_-VASc score was significantly correlated with a number of diseased vessels and associated with coronary artery disease severity.^7^ A total of 252 consecutive patients with non-ST segment elevation myocardial infarction (non-STEMI) were evaluated by Tasolar et al., and the CHA_2_DS_2_-VASc score was related with a higher Syntax score.^13^

However, to the best of our knowledge, the association between the CHA_2_DS_2_-VASc score and residual coronary artery disease severity after performing percutaneous coronary intervention (PCI) has not been studied yet.

Approximately 40-65% of multivessel coronary artery disease is detected in acute coronary syndrome patients, and it is also a predictor of poorer prognosis.^14 , 15^ The residual Syntax score (rSS) is a grading system to determine the complexity and severity of coronary atherosclerosis after performing PCI for culprit lesion. It was firstly used and described through a post hoc analysis of the ACUITY (Acute Catheterization and Urgent Intervention Triage strategY) trial.^2^ High rSS (>8) was a strong independent predictor of unplanned revascularization, myocardial infarction (MI), cardiac and 1-year mortality in 2686 patients with moderate-high risk acute coronary syndrome undergoing PCI.^2^ Supporting this, Loutfi et al. showed that lower rSS (a score of 8 or below) is associated with the reduction in 1 year of major adverse cardiac and cerebrovascular events (MACCE), death, MI, cerebrovascular accident and repeated revascularization in STEMI patients.^16^

An unexpected result of the substudy group of the COURAGE trial (clinical outcomes utilizing revascularization and aggressive drug evaluation) revealed that the extent and severity of the anatomic obstruction of coronary arteries had a more predictive value on MI and death compared to the degree of ischemia.^17^ It reflects the prognostic value of coronary atherosclerotic burden on adverse clinical outcomes. Thus, the importance of the residual coronary artery disease severity is revealed.

To the best of our knowledge, we also demonstrated the association of the CHA_2_DS_2_-VASc score and residual Syntax score (rSS) for the first time in STEMI patients who underwent primary PCI. It may be the reason for increased adverse cardiovascular outcomes in ST segment elevation myocardial infarction (STEMI) patients with a higher CHA_2_DS_2_-VASc score. However, large scaled studies are needed for future investigations, especially focused on clinical events.

### Study limitations

A relatively small sample size was the major limitation of our study. Lack of data about clinical outcomes and prognosis was the other major limitation. Some risk factors can be modified with lifestyle changes and medical therapy. However, this study was inadequate to demonstrate the effect of modified factors on clinical results due to the retrospective design of the study.

## Conclusion

The CHA_2_DS_2_-VASc score has a predictive value on rSS in patients with STEMI. The CHA_2_DS_2_-VASc score was also an independent predictor of higher rSS. Additionally, this score was positively correlated with coronary atherosclerotic burden.
